# Postpubertal Assessment of Treatment Timing in Class II Malocclusion Treated with Twin Block Followed by Fixed Appliances: A Retrospective Observational Study

**DOI:** 10.3390/jcm15093414

**Published:** 2026-04-29

**Authors:** Agnese Bonanno, Francesco Caroccia, Ramona Teodora Statie, Veronica Giuntini, Lorenzo Franchi

**Affiliations:** Graduate Orthodontic Program, Department of Experimental and Clinical Medicine, Università degli Studi di Firenze, Via del Ponte di Mezzo 46–48, 50127 Firenze, Italy; agnese.bonanno@unifi.it (A.B.); ramona.statie@edu.unifi.it (R.T.S.); veronica.giuntini@unifi.it (V.G.)

**Keywords:** cephalometrics, class II malocclusion, functional jaw orthopedics, timing, Twin Block, fixed appliances, orthodontic functional appliances

## Abstract

**Objectives:** To evaluate the role of treatment timing in the management of Class II malocclusions with mandibular retrusion using Twin Block (TB) followed by fixed appliances (FAs). **Methods:** Forty-one Caucasian patients (22 females and 19 males) with Class II malocclusion treated consecutively with TB and FA were selected from the Orthodontic Clinic of the Careggi University Hospital, Florence, Italy and from a sample treated by a private practitioner in Auckland, New Zealand. According to the Cervical Vertebral Maturation (CVM) method, the subjects were divided into two groups: an early treated group (ETG) including 21 patients (mean age 10.8 ± 2.1 years) who began treatment before the pubertal growth peak (CS1–CS2), and a late treated group (LTG) including 20 patients (mean age 12.4 ± 1.1 years) treated at the growth peak (CS3–CS4). Cephalometric skeletal, dento-alveolar and soft tissue parameters were evaluated before treatment with TB (T0) and at the mid-term observation (at a postpubertal stage CS4–CS6) after FA (T1). Independent samples *t*-tests were performed to compare intergroup differences at T0 and T1 while Fisher’s exact test was used to assess differences for gender. **Results:** At T0, the groups showed statistically significant differences in mandibular dimensions, in accordance with the different age distribution. At T1, significant differences between ETG and LTG were observed in Co-Gn (5.0 mm, *p* = 0.048, 95% Confidence Interval (CI) −9.8 mm; −0.1 mm), Co-Go (3.7, *p* = 0.009, 95%CI −6.3 mm; −0.9 mm), and Pg′-TVL SN10 (2.7 mm, *p* = 0.039, 95%CI −5.2 mm; −0.1 mm). **Conclusions:** Class II treatment with TB and FA confirmed that including the pubertal phase in the functional-orthopedic treatment led to more favorable mandibular growth and chin projection when evaluated at a postpubertal observation.

## 1. Introduction

Class II malocclusion is typically characterized by increased overjet and mandibular retrusion [1]. Therefore, in growing patients, the primary objective of Class II functional jaw orthopedics is to promote mandibular growth and correct the underlying skeletal discrepancy. This orthopedic approach is based on the ability to stimulate mandibular growth through forward mandibular displacement, which is achieved using functional appliances [2]. Several functional appliances, both removable and fixed, have been proposed to induce forward mandibular posturing.

Among the removable functional appliance, Twin Block (TB) is one of the most widely used and it is supported by a substantial body of scientific evidence [3,4,5,6,7,8]. Developed by William J. Clark, the Twin Block consists of two separate plates for the upper and lower arches, equipped with complementary inclined planes that interlock during occlusion [9]. Specifically, the posterior blocks of the upper plate articulate with the anterior blocks of the lower plate, forcing the mandible to advance upon closure. The efficacy of the appliance relies on its ability to influence mandibular growth through voluntary muscular activation, encouraging the patient to actively maintain the mandible in a forward position. Despite its widespread clinical use, the role of functional appliances in effectively enhancing mandibular growth remains controversial. In 2015, D’Antò et al. summarized the available literature on functional orthopedic treatment of Class II malocclusion in a systematic review of systematic reviews [10]. The authors concluded that there is some evidence of increased mandibular length following treatment with various functional appliances; however, these effects appear to be limited to short-term observations. In addition, evidence supporting favorable soft tissue changes was found to be insufficient.

One of the main reasons for the lack of strong evidence in this field lies in the methodological limitations of the primary studies included in systematic reviews. These limitations include the absence of matched untreated control groups, small sample sizes, heterogeneity in cephalometric analyses, and variability in treatment timing [10]. Indeed, the effectiveness of functional orthopedic therapy depends not only on the type of appliance used but also on the patient’s growth potential at the time of treatment, which can be assessed using skeletal maturity indicators [11].

Concurrently with the systematic review by D’Antò et al. [10], another systematic review focusing specifically on the influence of treatment timing in Class II malocclusion treated with removable functional appliances was published but was not included in the former analysis [12]. Perinetti et al. [12] reported that, despite the limited quality of the available studies, removable functional appliances were effective in producing mandibular skeletal changes when treatment was initiated during the pubertal growth peak.

However, these conclusions are based exclusively on short-term observations, typically recorded at the end of the functional treatment phase. In most clinical protocols, the functional phase is followed by a refinement phase, during which occlusion is finalized using fixed appliances (FAs) or aligners. To date, no study using the Twin Block as a functional appliance has investigated the influence of treatment timing on treatment outcomes in the medium term, that is, after completion of the fixed orthodontic phase and at a postpubertal stage.

The aim of the present study, therefore, was to evaluate the skeletal, dentoalveolar, and soft tissue changes produced by Twin Block followed by fixed appliance therapy in two groups of patients with Class II malocclusion treated at different stages of skeletal maturation (before and during the pubertal growth peak), in order to determine the optimal timing of treatment at a postpubertal observation.

## 2. Materials and Methods

### 2.1. Study Design, Setting and Participants

This retrospective, observational multicenter study was written according to the STROBE guidelines [13] (Supplementary Table S1). During the preparation of the manuscript, ChatGPT (version GPT-5.3) was used to assist with English language editing. The study was conducted in accordance with the Declaration of Helsinki and approved by the Regional Pediatric Ethics Committee of Tuscany (Florence, Italy; protocol number 156/2025; date of approval: 15 July 2025).

Sample size was calculated for an independent-samples *t* test to detect a difference of 2.0 degrees in the primary outcome variable (Pg′-TVL), assuming a standard deviation of 2.0 degrees, based on previously published data [7]. An alpha level of 0.05 and a statistical power of 0.80 were adopted, resulting in a required sample size of 17 subjects per group. The sample size calculation was based on the primary outcome variable (Pg′-TVL SN10), selected for its clinical relevance in assessing soft tissue chin projection. Other cephalometric variables were considered secondary outcomes, and the study was not specifically powered to detect differences in these parameters.

Cephalometric records of 41 Caucasian patients (22 females, 19 males) with Class II division 1 malocclusion treated consecutively with the TB followed by FA were collected. Class II subjects were retrieved from the records of patients treated at the Orthodontic Clinic of the University Hospital of Careggi, Florence, Italy during the period from January 2007 to September 2017 (23 subjects; 10 females, 13 males), and from a sample of patients kindly provided by Dr. Forbes Leishman treated at his private practice in Auckland, New Zealand (18 subjects; 12 females, 6 males).

In both samples, the TB appliances followed the original design proposed by Clark [9]. The bite registration procedure, as well as the choice between single-step or stepwise mandibular advancement, was determined based on the initial overjet, as previously described [14]. According to Clark’s recommendations, the acrylic on the posterior portion of the maxillary bite blocks was selectively trimmed in patients presenting with reduced lower anterior facial height and a pronounced curve of Spee [9]. Patients were instructed to wear the TB appliance for approximately 14 h per day (during nighttime and for several hours during the day, excluding school time, meals, and certain sports activities). The mean duration of TB treatment was 1.1 years, after which patients were immediately transitioned to 0.022″ slot preadjusted fixed appliances.

Inclusion criteria comprised the presence of Class II malocclusion with a clinical indication for treatment with TB followed by FA, defined as an improvement in facial profile following the Frankel maneuver [15]. Additionally, the availability of two lateral cephalograms for each subject was required: one obtained before the start of TB therapy (T0) and one immediately after completion of FA therapy (T1). Exclusion criteria included cleft lip and/or palate, craniofacial syndromes, incomplete eruption of the maxillary central incisors, or dental agenesis.

Patients were divided into two groups based on their skeletal maturity at the start of treatment, evaluated by means of the Cervical Vertebral Maturation (CVM) method on the initial cephalogram (T0) [16]. The early treated group (ETG) consisted of 21 subjects (10 females, 11 males; average age 10.8 ± 2.1 years) presenting with either cervical stage 1 or 2 (CS1, CS2) in CVM (i.e., before the onset of the pubertal growth spurt). The late treated group (LTG) consisted of 20 subjects (12 females and 8 males; average age 12.4 ± 1.1 years) presenting with CS3 or CS4. At T1, all patients of both groups had to show a postpubertal stage (CS4–CS6). CVM staging was performed by an expert examiner (AB).

### 2.2. Cephalometric Analysis

**Figure 1:**
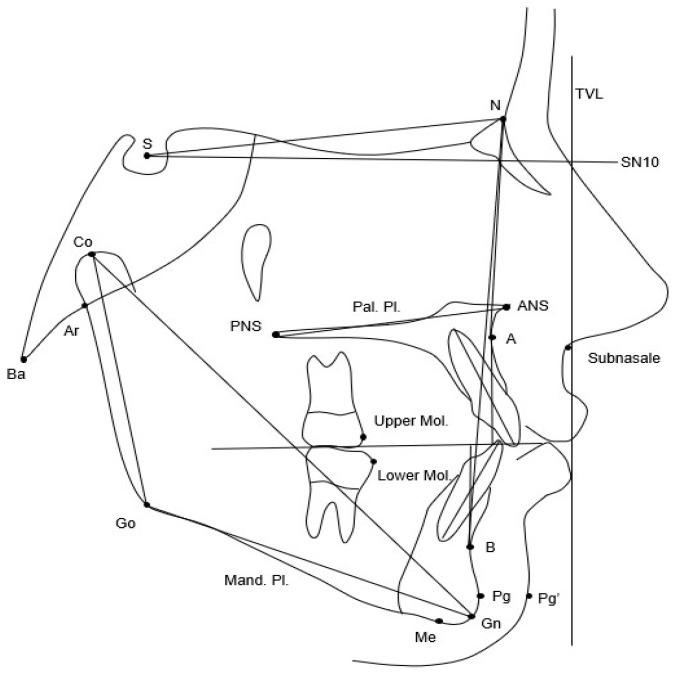
Cephalometric parameters measured at T0 and at T1. *S* sella, *N* nasion, *PNS* posterior nasal spine, *ANS* anterior nasal spine, *Pal.* palatal, *Pl.* plane, *Mand.* mandibular, *SN10* a plane passing through S and inclined 10° downward to SN, *TVL* true vertical line (perpendicular SN10 and passing through point subnasale), *Go* Gonion, *Co* Condilion, *Me* Menton, *Gn* Gnathion, *Pg*′ soft tissue Pogonion, *A* A point, *B* B point, *Upper Mol.* mesial point of upper molar, *Lower Mol.* mesial point of lower molar, *Ba* Basion, *Ar* Articulare.

Lateral cephalograms of both groups at T0 and at T1 were standardized as to 0% magnification factor (life size) and analyzed by one investigator (AB). A customized digitization regimen (Viewbox, version 4.1.0.12, dHAL Software, Kifissia, Greece) was created and used for cephalometric evaluation. A custom cephalometric analysis composed by 19 variables (9 linear and 10 angular) was performed (Figure 1).

To evaluate the position of the soft tissue chin, the distance from the soft tissue pogonion (Pg′) to a true vertical line (TVL) was traced. This TVL was defined as a line passing through the subnasale point and perpendicular to a reference horizontal line passing through the Sella point (S), inclined 10 degrees downward relative to the SN plane. This reference line has been shown to be assimilable to a true horizontal line, as reported by Gonca et al. [17]. This horizontal reference plane was used instead of the Frankfurt plane to overcome the possible difficulties in locating the Porion and Orbitale points necessary for defining the Frankfurt plane.

### 2.3. Method Error

Fifteen cephalograms were selected randomly from the total sample and re-digitized after a wash-out period of 2 weeks by the same operator (AB). Intraobserver reproducibility was assessed with the intraclass correlation coefficient (ICC) for the CVM staging and cephalometric variables.

### 2.4. Statistical Analysis

Descriptive statistics were performed using means and standard deviations for quantitative variables and frequency and percentage for qualitative variables.

Independent sample *t*-tests were performed for intergroup statistical comparisons for age and all cephalometric variables at baseline (T0) and at the end of FA therapy (T1). Fisher’s exact test was used to assess differences for gender. All statistical analyses were performed using statistical software packages (IBM SPSS Statistics, ver. 29.0, Armonk, NY, USA and MedCalc version 19.6.4, MedCalc Software Ltd., Ostend, Belgium).

## 3. Results

Patients were divided into two groups based on their skeletal maturity at the start of treatment, evaluated by means of the Cervical Vertebral Maturation (CVM) method on the initial cephalogram (T0) [16]. The early treated group (ETG) consisted of 21 subjects (10 females, 11 males; mean age 10.8 ± 2.1 years) presenting with either cervical stage 1 or 2 (CS1, CS2) in CVM (i.e., before the onset of the pubertal growth spurt). The late treated group (LTG) consisted of 20 subjects (12 females e 8 males; mean age 12.4 ± 1.1 years) presenting with CS3 or CS4. At T1, all patients of both groups had to show a postpubertal stage (CS4–CS6). Mean age at T1 for ETG was 15.2 ± 1.4 years and for LTG it was 15.5 ± 1.9 years.

All ICC for the cephalometric variables were classified as “almost perfect”, ranging, for linear measurements, from a minimum of 0.83 for the Wits appraisal to a maximum of 0.99 for N–Me and overjet; and, for angular variables, from a minimum of 0.86 for ANB to a maximum of 0.99 for SN-Mand. Pl. and Pal. Pl.-Mand.Pl. (Table 1). Intraobserver reproducibility for CVM was also “almost perfect” with a value for ICC of 0.87 (Table 1).

Fisher’s exact test showed no statistically significant difference in gender distribution between the two groups (*p* = 0.771). A significant difference in age distribution was observed at T0 (10.8 ± 2.1 years in the ETG vs. 12.4 ± 1.1 years in the LTG; *p* = 0.003), whereas no significant difference was found at T1 (15.2 ± 1.5 years and 15.5 ± 1.9 years, respectively; *p* = 0.664) (Table 2 and Table 3).

The mean duration of therapy, calculated as the interval between T0 and T1, was not statistically significant between ETG (4.4 ± 2.4 years) and LTG (3.0 ± 2.0 years) (*p* = 0.05).

At baseline (T0), comparison between the ETG and LTG revealed statistically significant differences in several variables: SNB (73.8 ± 2.8 vs. 75.8 ± 2.7; *p* = 0.028), with a mean difference of 2.0°; Co-Gn (103.8 ± 9.6 mm vs. 111.5 ± 7.1 mm; *p* = 0.006), with a mean difference of 7.7 mm; Co-Go (50.3 ± 5.5 mm vs. 54.0 ± 4.2 mm; *p* = 0.020), with a mean difference of 3.7 mm; and overbite (3.2 ± 2.3 mm vs. 5.4 ± 2.0 mm; *p* = 0.020), with a mean difference of 2.2 mm (Table 2). These differences are most likely attributable to the age discrepancy between the two groups at T0.

Descriptive statistics and comparisons between the early treated group (ETG) and the late treated group (LTG) at the end of fixed appliance treatment (T1) are reported in Table 3. At T1, statistically significant differences between the ETG and LTG were observed for several cephalometric variables (Table 3): Co-Gn (115.7 ± 7.1 mm vs. 120.7 ± 8.3 mm; *p* = 0.048), with a mean difference of 5.0 mm; Co-Go (56.7 ± 4.5 mm vs. 60.4 ± 4.0 mm; *p* = 0.009), with a mean difference of 3.7 mm; and Pg′-TVL SN10 (−11.6 ± 3.4 mm vs. −8.9 ± 4.6 mm; *p* = 0.039), with a mean difference of 2.7 mm. All these differences were also clinically relevant, as they exceeded the threshold of 2 mm.

## 4. Discussion

The present retrospective observational study investigated the role of treatment timing in the management of Class II malocclusion due to mandibular retrusion using Twin Block (TB) followed by fixed appliances (FAs). The primary aim was to evaluate dentoskeletal and soft tissue changes at a medium-term observation, defined as immediately after removal of a FA in a postpubertal developmental phase, by comparing subjects treated before the pubertal growth peak (early treated group, ETG) with those treated during the pubertal growth peak (late treated group, LTG). To date, the literature lacks studies specifically addressing medium-term outcomes of Class II treatment with TB in relation to treatment timing, making the present investigation particularly relevant.

A previous investigation by Baccetti et al. evaluated the influence of treatment timing on Class II correction with TB using a short-term observation period [3]. The authors reported more favorable skeletal mandibular effects when TB therapy was initiated during or immediately after the onset of the pubertal growth peak compared with early prepubertal treatment. Although the study design was similar to the present investigation, the observation period was limited to the end of the functional phase. Comparable conclusions were reported by Singh et al., who observed greater mandibular effects following approximately two years of TB wear when treatment was initiated during puberty [18].

The results of the present study confirm, at a medium-term observation, the decisive role of treatment timing in the orthopedic management of Class II malocclusion due to mandibular retrusion. Comparisons between the ETG and LTG at T1 revealed statistically and clinically significant differences in mandibular skeletal parameters, including Co-Gn (115.7 ± 7.1 mm in ETG vs. 120.7 ± 8.3 mm in LTG; *p* = 0.048) and Co-Go (56.7 ± 4.5 mm vs. 60.4 ± 4.0 mm; *p* = 0.009), with more favorable outcomes observed in patients treated during the pubertal growth peak. These findings suggest that the skeletal effects reported in short-term studies are not transient but remain evident after completion of comprehensive orthodontic treatment. These results corroborate the findings of Baccetti et al. [3], extending their observations beyond the functional phase and demonstrating stability of the skeletal effects into the postpubertal period. Further support for the critical role of treatment timing is provided by the systematic review by Perinetti et al., which demonstrated that clinically relevant skeletal effects of removable functional appliances occur almost exclusively when treatment is initiated during the pubertal growth phase [12]. In that review, pubertal patients exhibited a mean mandibular length increase of approximately 3.0 mm compared with untreated controls, whereas prepubertal patients showed a much smaller increase of about 1.0 mm. Similarly, an increase in ramus height of 2.2 mm was reported only in pubertal subjects, indicating a limited orthopedic response when treatment is initiated before the pubertal growth peak.

The positive skeletal effects on mandibular stimulation observed in our study may appear to contrast with the findings of the UK multicenter randomized controlled trial (RCT) conducted by O’Brien et al. [4] and three other contemporary RCTs [19,20,21], which reported that TB therapy primarily produced dentoalveolar rather than skeletal changes. However, these findings further underscore the importance of appropriate treatment timing, as in those studies treatment was initiated before the pubertal growth spurt.

Previous studies have also investigated the influence of treatment timing on the effects of functional appliances followed by FAs [22,23]. However, none of these studies specifically evaluated TB therapy. In a retrospective controlled long-term study using either the Bionator or Activator, Pavoni et al. reported greater mandibular growth enhancement and chin advancement in patients treated during puberty, whereas subjects treated before puberty exhibited predominantly dentoalveolar changes [23]. These findings are consistent with the results of the present study and support the concept that skeletal responsiveness to functional therapy is strongly growth dependent.

In addition to skeletal changes, the present study demonstrated a significant impact of functional therapy on facial soft tissues. Patients treated during the pubertal growth peak showed a significantly greater advancement of the soft tissue Pogonion, as indicated by a true vertical line passing through soft tissue Subnasale (Pg′-TVL SN10 measurement), with a mean difference of 2.7 mm compared with early-treated patients. This finding suggests that true mandibular advancement achieved during puberty is accompanied by clinically relevant improvements in facial profile aesthetics.

These results are consistent with those reported by Khoja et al., who found that TB treatment initiated during the pubertal growth peak resulted in significant increases in mandibular length and SNB angle, as well as a reduction in the ANB angle, confirming effective mandibular advancement [24]. Moreover, pubertal patients exhibited clinically relevant advancement of the soft tissue pogonion, whereas early-treated subjects showed more limited and less consistent soft tissue responses.

With respect to the type of functional appliance, several comparative studies have demonstrated the effectiveness of the TB in promoting skeletal and soft tissue changes. Cretella Lombardo et al. reported that TB was more effective than Invisalign^®^ mandibular advancement in producing soft tissue chin advancement [7]. Pacha et al. observed that Hanks Herbst was more efficient than TB in reducing overjet but with greater deterioration in oral health-related quality of life and more routine and emergency visits [25]. Additionally, a recent randomized controlled trial comparing TB with FA alone concluded that TB therapy produced more favorable skeletal effects, whereas FA treatment primarily affected the dentoalveolar component [26]. A CBCT study further demonstrated greater mandibular condylar remodelling in patients treated with TB compared with fixed functional appliances such as PowerScope™ [27]. Collectively, these findings confirm the efficacy of the TB in stimulating mandibular growth and improving facial soft tissue profile, supporting its suitability for studies investigating treatment timing at a medium-term observation.

In conclusion, the findings of the present study indicate that treatment of Class II malocclusion due to mandibular retrusion is significantly more effective when initiated during the pubertal growth peak. The greater increases in mandibular length, ramus height, and advancement of the soft tissue Pogonion observed in pubertally treated patients highlight the importance of accurate assessment of skeletal maturity and individualized treatment timing. Such an approach maximizes orthopedic efficacy and facilitates the achievement of stable functional and aesthetic outcomes.

It should be noted that the sample size calculation was based on the primary outcome variable (Pg′-TVL SN10). Therefore, the study was specifically powered for this endpoint, whereas the other cephalometric variables should be considered secondary outcomes and interpreted with appropriate caution. In this context, the statistically significant differences observed for Co-Gn and Co-Go should be regarded as supportive of the main findings rather than as independent confirmatory evidence.

The findings of the present study are primarily generalizable to growing Caucasian patients with Class II malocclusion due to mandibular retrusion treated with Twin Block followed by fixed appliances in comparable clinical settings. Caution is warranted when extrapolating these results to different ethnic populations, growth patterns, or alternative functional appliances. Nonetheless, the consecutive inclusion of patients and postpubertal assessment enhance the applicability of the findings to routine orthodontic practice.

Some limitations of the present study should be acknowledged, including the relatively small sample size, the retrospective design and the presence of only Caucasian patients. These factors may affect the generalizability and statistical power of the findings. One aspect that warrants particular attention is the absence of an untreated control group, which would have allowed a clearer distinction between the true effects of treatment and changes attributable to physiological craniofacial growth. However, medium-term observational studies involving prepubertal subjects over extended follow-up periods—up to 11 years in the present investigation—are inherently challenging due to difficulties in long-term patient retention. Consequently, the implementation of prospective designs or inclusion of untreated control groups is often impractical. Another limitation of the study is that, although removable appliances were used, no objective methods were employed to monitor patient compliance or actual daily wear time.

A statistically significant difference in chronological age between the two groups was observed at T0, which represents a potential confounding factor in growth-related studies. However, this difference is intrinsically linked to the study design, as group allocation was based on skeletal maturation stages rather than chronological age. The baseline differences in mandibular dimensions are therefore consistent with physiological growth variations. Importantly, both groups reached a comparable postpubertal stage at T1, with no significant differences in age, thereby reducing the impact of baseline discrepancies on the interpretation of treatment outcomes. Nonetheless, the potential influence of baseline age differences should be considered when interpreting the findings.

All cephalometric measurements were performed by a single calibrated examiner, and intra-examiner reliability was assessed, showing excellent reproducibility. Although inter-examiner reliability was not evaluated, this was consistent with the study design, as measurements were not distributed across multiple operators. However, the lack of inter-examiner assessment may limit the generalizability of the measurement protocol and should be considered when interpreting the findings.

Although no statistically significant difference in treatment duration was observed between the two groups, the relatively large variability within each group should be acknowledged. This variability likely reflects individual differences in growth potential, treatment response, and clinical management. While it does not appear to have introduced a systematic bias between groups, it may have contributed to increased dispersion of the outcomes and should be considered when interpreting the findings.

Future research should focus on prospective controlled trials and on enlarging retrospective cohorts through the inclusion of additional eligible cases, as well as on continued follow-up of the current sample.

## 5. Conclusions

Postpubertal observations suggest that Class II treatment with Twin Block followed by fixed appliances is more effective in promoting mandibular growth and chin projection when functional therapy is initiated during the pubertal growth phase.

## Figures and Tables

**Table 1:** Intra-examiner reliability of measurements assessed using Intraclass Correlation Coefficient (ICC).

Variable	ICC	95% Confidence Interval	
		Lower	Upper
N-S-Ba (°)	0.90	0.72	0.73
SNA (°)	0.95	0.86	0.98
SNB (°)	0.98	0.93	0.99
ANB (°)	0.86	0.64	0.95
Wits (mm)	0.83	0.58	0.94
SN-Pal. Pl. (°)	0.93	0.81	0.98
SN-Mand. Pl. (°)	0.99	0.97	1.00
Pal. Pl.-Mand. Pl. (°)	0.99	0.96	1.00
Co-Gn (mm)	0.95	0.85	0.98
Co-Go (mm)	0.95	0.87	0.98
N-Me (mm)	0.99	0.96	1.00
ANS-Me (mm)	0.97	0.90	0.99
Co-Go-Me (°)	0.95	0.82	0.98
Overjet (mm)	0.99	0.96	1.00
Overbite (mm)	0.97	0.92	0.99
Molar rel. (mm)	0.93	0.79	0.97
Upper Inc.-Pal. Pl. (°)	0.94	0.83	0.98
Lower Inc.-Mand. Pl. (°)	0.95	0.86	0.98
Pg′-TVL SN10 (mm)	0.97	0.91	0.99
CVM stage	0.87	0.77	0.96

Pal. Palatal; Mand. Mandibular; Pl. Plane; Rel. Relationship; Inc. Incisor.

**Table 2:** Descriptive statistics and intergroup comparisons at T0. Level of significance was set at *p* < 0.05.

Variables	ETG T0 n. = 21		LTG T0 n. = 20		Mean Diff.	*p* Value	95% Confidence Interval	
	Mean	SD	Mean	SD			Lower	Upper
Age (years)	10.8	2.1	12.4	1.1	1.6	0.003	−2.7	−0.6
N-S-Ba (°)	133.6	4.4	131.2	5.1	−2.4	0.116	−0.6	5.4
SNA (°)	80.7	2.8	82.2	2.6	1.5	0.096	−3.2	0.3
SNB (°)	73.8	2.8	75.8	2.7	2.0	0.028	−3.7	0.2
ANB (°)	6.9	1.3	6.4	2.0	−0.5	0.345	−0.6	1.5
Wits (mm)	5.7	1.8	6.0	3.1	0.3	0.710	−1.9	1.3
SN-Pal. Pl. (°)	9.1	2.8	7.5	3.3	−1.6	0.099	−0.3	3.5
SN-Mand. Pl. (°)	32.7	5.0	30.1	5.6	−2.6	0.130	−0.8	5.9
Pal. Pl.-Mand. Pl. (°)	23.6	5.1	22.6	6.8	−1.0	0.625	−2.9	4.7
Co-Gn (mm)	103.8	9.6	111.5	7.1	7.7	0.006	−13.0	−2.3
Co-Go (mm)	50.3	5.5	54.0	4.2	3.7	0.020	−6.9	−0.6
ANS-Me (mm)	61.1	6.4	65.0	7.0	3.9	0.070	−8.1	0.3
Co-Go-Me (°)	125.0	5.7	122.2	5.6	−2.8	0.112	−0.7	6.4
Overjet (mm)	8.6	2.1	9.4	3.3	0.8	0.327	−2.6	0.9
Overbite (mm)	3.2	2.3	5.4	2.0	2.2	0.002	−3.6	−0.8
Molar rel.(mm)	−2.5	1.6	−2.2	1.9	0.3	0.648	−1.4	0.9
Upper Inc.–Pal. Pl. (°)	114.8	8.1	115.5	5.3	0.7	0.725	−5.1	3.6
Lower Inc.–Mand. Pl. (°)	99.2	5.5	98.0	6.0	−1.2	0.496	−2.4	4.9
Pg′-TVL SN10 (mm)	−13.0	2.9	−11.1	3.5	1.9	0.058	−4.0	0.1

n. Number; SD Standard Deviation; Diff. Difference; Pal. Palatal; Mand. Mandibular; Pl. Plane; Rel. Relationship; Inc. Incisor.

**Table 3:** Descriptive statistics and intergroup comparisons at T1. Level of significance was set at *p* < 0.05.

Variables	ETG T1 n. = 21		LTG T1 n. = 20		Mean Diff.	*p* Value	95% Confidence Interval	
	Mean	SD	Mean	SD			Lower	Upper
Age (years)	15.2	1.5	15.5	1.9	0.3	0.664	−1.3	0.8
N-S-Ba (°)	133.4	5.5	130.2	6.2	−3.2	0.088	−0.5	6.9
SNA (°)	80.7	4.1	81.6	2.9	0.9	0.459	−3.1	1.4
SNB (°)	76.3	3.7	77.9	3.0	1.6	0.147	−3.7	0.6
ANB (°)	4.4	1.7	3.7	1.9	−0.7	0.210	−0.4	1.9
Wits (mm)	2.6	2.7	0.8	3.8	−1.8	0.092	−0.3	3.8
SN—Pal. Pl. (°)	9.4	4.1	7.7	2.9	−1.7	0.135	−0.6	4.0
SN—Mand. Pl. (°)	31.8	4.9	29.7	5.8	−2.1	0.225	−1.3	5.5
Pal. Pl.—Mand. Pl. (°)	22.4	5.2	22.1	6.9	−0.3	0.860	−3.5	4.2
Co-Gn (mm)	115.7	7.1	120.7	8.3	5.0	0.048	−9.8	−0.1
Co-Go (mm)	56.7	4.5	60.4	4.0	3.7	0.009	−6.3	−0.9
ANS-Me (mm)	67.4	6.1	69.9	7.9	2.5	0.267	−6.9	2.0
Co-Go-Me (deg)	123.7	5.6	122.1	5.3	−1.6	0.360	−1.9	5.1
Overjet (mm)	2.8	0.8	2.4	1.1	−0.4	0.326	−0.3	0.9
Overbite (mm)	2.1	1.1	1.4	1.6	−0.7	0.097	−0.1	1.6
Molar rel.(mm)	2.0	0.9	2.5	1.1	0.5	0.201	−1.1	0.2
Upper Inc.–Pal. Pl. (°)	112.2	5.6	111.7	5.6	−0.5	0.785	−3.1	4.0
Lower Inc.–Mand. Pl. (°)	102.1	5.9	102.8	8.8	0.7	0.758	−5.4	4.0
Pg′-TVL SN10 (mm)	−11.6	3.4	−8.9	4.6	2.7	0.039	−5.2	−0.1

n. Number; SD Standard Deviation; Diff. Difference; Pal. Palatal; Mand. Mandibular; Pl. Plane; Rel. Relationship; Inc. Incisor.

## Data Availability

The data presented in this study are available upon request from the corresponding authors.

## Supplementary Materials

The following supporting information can be downloaded at: https://www.mdpi.com/article/10.3390/jcm15093414/s1, Table S1: STROBE Statement—checklist of items that should be included in reports of observational studies.

## Abbreviations

The following abbreviations are used in this manuscript:

TB	Twin Block
FA	Fixed appliance
CVM	Cervical vertebral maturation
ETG	Early treated group
LTG	Late treated group
RCT	randomized controlled trial
